# Suggestions Concerning Long Life

**Published:** 1881-08

**Authors:** 


### SUGGESTIONS CONCERNING LONG LIFE.

If any one could furnish the world with a medicine that would insure a long life, there is no end to the demand he would have for his drug. The *Herald of Health* thinks he would need many factories to make it, and many banks to hold the money he would receive. Fortunately, there is no such medicine, and so the world will have to get along in some other way.

Some time ago the French Government sent a circular letter to all the districts of that country to collect information as to those conditions of life which seemed to favor longevity. The replies were very interesting, but on the whole rather monotonous; and the general result was that longevity is promoted by great sobriety, regular labor, especially in the open air, short of excessive fatigue, easy hours, a well-off condition, a philosophical mind in meeting troubles, not too much intellect, and a domestic life. The value of marriage was universally admitted, and long-lived parents were also found an important factor. A healthy climate and good water were mentioned. All this agrees with common sense, unless the idea that the intellect is a hinderance to longevity be considered unreasonable, and we know that some of the most intellectual men have lived to a great age.

